# Primary Cutaneous Histiocytic Sarcoma: A Rare Case

**DOI:** 10.7759/cureus.89241

**Published:** 2025-08-02

**Authors:** Nektarios Ntalakos, Maria Arnaouti

**Affiliations:** 1 Department of Pathology, Saint Savvas Anticancer Hospital of Athens, Athens, GRC

**Keywords:** dermatopathology, hematopathology, histiocytic cell neoplasm, histiocytic neoplasms, histiocytic sarcoma

## Abstract

Histiocytic sarcoma is a rare malignancy composed of neoplastic cells that resemble macrophages in morphology and immunoprofile. It manifests in lymph nodes or extranodal sites, with the majority occurring in the gastrointestinal tract, central nervous system, spleen, skin, and soft tissues. Clinical manifestations depend on organ involvement, with systemic symptoms, including fever, fatigue, night sweats, weight loss, and weakness. A significant proportion of patients are diagnosed at an advanced stage, which is associated with high mortality rates. In this report, we highlight the case of an 80-year-old who displayed a subcutaneous forearm lesion. Histological evaluation confirmed the diagnosis of cutaneous histiocytic sarcoma, with tumor cells expressing CD163 and CD68. A multidisciplinary team decided on supplementary adjuvant radiotherapy, considering the unifocal presentation and complete surgical excision of the lesion. The patient remains disease-free at one-year follow-up. This case emphasizes that a unifocal and localized manifestation of the malignancy has a more favorable outcome. Although treatment protocols have not yet been standardized, recent advances in genomic profiling may offer opportunities for targeted therapies.

## Introduction

Histiocytic sarcoma is a rare malignancy composed of neoplastic cells that resemble macrophages in morphology and immunoprofile [1]. It comprises 1% of hematolymphoid tumors [2], manifesting most frequently in adults [2], with a male predominance being mentioned in some series [3]. The usual age demographic being reported consists of patients in their 40s and 50s [3], with infants and children also being affected [2]. Histiocytic sarcoma presents in lymph nodes or extranodal sites [4], with the majority of cases occurring in the gastrointestinal tract, central nervous system, spleen, skin, and soft tissues [1,3]. Its etiology and pathogenesis remain unknown [3]. A few cases have been associated with mediastinal germ cell tumors [1], mostly malignant teratoma [3], while a subset of cases occurred in association with other hematological malignancies [3], including B-cell lymphomas [2] and myelodysplasia and leukemia [3]. A significant proportion of patients are diagnosed at an advanced stage, with mortality rates reported to be between 60% and 80% [3]. However, cases with clinically localized and resectable tumours had a favorable outcome [1,2].

## Case presentation

An 80-year-old male, with unremarkable medical history, presented to the Orthopedic Outpatient Clinic with localized pain in his left forearm. Physical examination revealed a small palpable subcutaneous nodule. The lesion was surgically excised and sent to the Pathology Department for evaluation. Gross examination revealed a soft, pale-white, and well-circumscribed dermal lesion measuring 40 mm.

**Figure 1:**
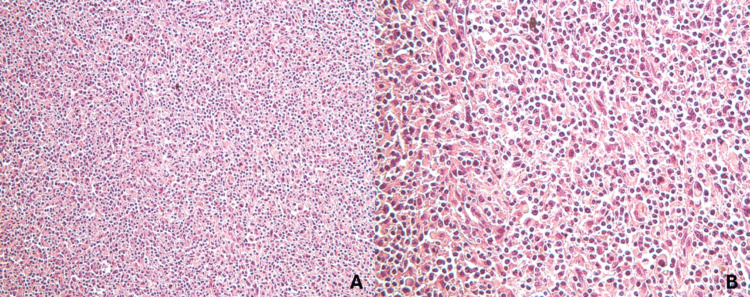
Histopathological imaging of histiocytic sarcoma A: H&E stain (X200). B: H&E stain (X400)

**Figure 2:**
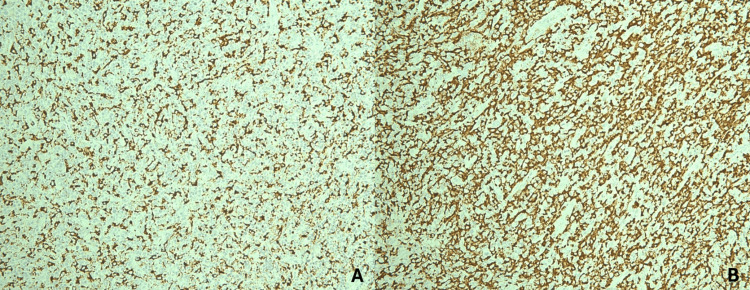
Immunohistochemical analysis of histiocytic sarcoma A: Neoplastic cells positive for CD68. B: Neoplastic cells positive for CD163

**Figure 3:**
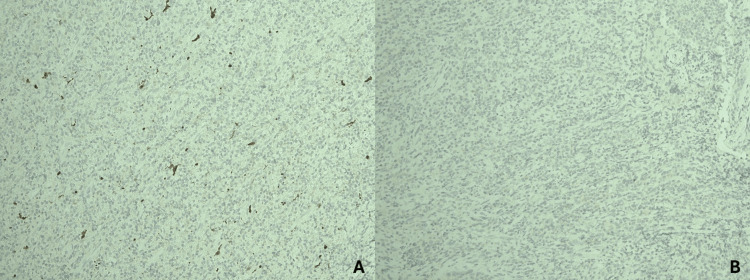
Immunohistochemical analysis of histiocytic sarcoma A: Neoplastic cells negative for S100. B: Neoplastic cells negative for cytokeratin AE1/AE3

Microscopically, the lesion consisted of large neoplastic cells with abundant eosinophilic cytoplasm, eccentrically placed nuclei, and increased mitotic activity (Figure 1). The tumor was in close proximity to the surgical resection margins. Immunohistochemistry showed strong positivity for CD163 and CD68, indicating a histiocytic lineage in the tumor cells. Weak staining for CD45 was observed, while markers including cytokeratin AE1/AE3, S-100, CD3, CD5, CD10, CD15, CD20, CD30, CD34, and CD56 were negative, supporting the diagnosis of cutaneous histiocytic sarcoma (Figures 2, 3). Due to the lack of formal staging guidelines and the localized nature of the disease, the American Joint Committee on Cancer (AJCC) TNM (tumor, nodes, and metastasis) staging system for soft tissue sarcomas was applied, and the tumor was classified as stage IA.

Following diagnosis and due to the rarity of this histopathologic entity, the specimen was referred to the Hematopathology Department for further review and the Genetics Laboratory for molecular profiling. PET-CT and extensive laboratory testing, including complete blood count, lactate dehydrogenase (LDH) levels, liver and renal function panels, C-reactive protein (CRP), and peripheral blood flow cytometry, were performed to exclude multifocal or systemic disease. A multidisciplinary team meeting was then initiated to determine appropriate treatment strategies. Considering the unifocal disease presentation and complete surgical excision, adjuvant radiotherapy was implemented to reduce the risk of recurrence. There are no standardized follow-up protocols for cutaneous histiocytic sarcoma. For the first two years, routine surveillance was planned, consisting of clinical examination, including skin and lymph node evaluation every three months, and PET-CT every six months. During years three to five, clinical examination will be performed every six months, with PET-CT reserved for cases with clinical suspicion or the presence of symptoms. After year five, an annual clinical examination will be recommended. At one-year follow-up, the patient remains disease-free.

## Discussion

Histiocytic sarcoma remains a rare and intriguing malignant entity, with clinical manifestations depending on organ involvement, unifocal or multifocal disease [1-4]. Systemic symptoms, including fever, fatigue, night sweats, weight loss, and weakness, are relatively common [1,2]. Physical findings consist of lymphadenopathy or manifestations of extranodal disease, such as skin lesions, intestinal obstruction, and hepatosplenomegaly [1,2]. Lytic lesions in bones and central nervous system lesions have also been described [2].

No standard treatment protocols have been developed, with surgery, radiotherapy, and systemic chemotherapy being implemented on a case-to-case basis [4]. Surgery followed by adjuvant radiotherapy is a well-documented course of action for unifocal disease [4]. Radical radiotherapy has also been executed as an alternative treatment for localized cases where surgery was contraindicated [4]. Combined systemic chemotherapy is being applied in patients with multifocal disease [4], considering no large prospective trials for patients with multifocal or systemic disease are being conducted [4]. A series of anthracycline-based and platinum-based regimens or ifosfamide-based regimens have been administered [4], with autologous or allogeneic hematopoietic stem cell transplantation proposed as salvage therapy [4].

On a molecular basis, alterations affecting the RAS/RAF/MAPK signaling cascade - such as KRAS, NRAS, BRAF, and others - are commonly reported in histiocytic sarcoma [1,5]. Mutations in tumour suppressor genes such as CDKN2A and TP53 are also common [1,5]. Less frequently described alterations include PI3K/AKT pathway gene mutations (PIK3R1, PIK3CA), CSF1R mutations, and BRAF and NTRK1 rearrangements [1,5].

Recent studies propose a molecular subclassification of histiocytic sarcoma based on the detection or lack of NF1/PTPN11 mutations [5]. The NF1/PTPN11 wild-type category had RAS/MAPK pathway activating mutations, involving KRAS, NRAS, BRAF, and MAP2K1 [5]. This subgroup also demonstrates immunoglobulin gene rearrangements and genetic alterations typically linked to B-cell neoplasms [5], further underscoring the association of histiocytic sarcoma with prior or metachronous lymphoma [1]. The NF1/PTPN11 mutated subgroup was characterized by loss of gene sets related to cellular proliferation and NF1 and PTPN11 loss-of-function mutations [5]. Except for SETD2, no other alterations in genes associated with B-cell lymphomas were noted [5]. This group is also associated with frequent involvement of the gastrointestinal tract [5]. These findings suggest that certain patients could potentially benefit from a more targeted therapeutic approach [5].

## Conclusions

Histiocytic sarcoma is an aggressive malignancy with distinct histopathological and immunohistochemical features. It is associated with a high mortality rate, with many patients diagnosed at an advanced stage. However, cases with unifocal and localized presentation have a more favorable outcome. Treatment protocols have not yet been standardized, but recent advances in genomic profiling may offer opportunities for targeted therapies.
